# Genetic diversity of symbiotic bacteria nodulating common bean (*Phaseolus vulgaris*) in western Kenya

**DOI:** 10.1371/journal.pone.0207403

**Published:** 2018-11-15

**Authors:** Fanuel Kawaka, Huxley Makonde, Mathews Dida, Peter Opala, Omwoyo Ombori, John Maingi, John Muoma

**Affiliations:** 1 Department of Applied Plant Sciences, Maseno University, Maseno, Kenya; 2 Department of Pure and Applied Sciences, Technical University of Mombasa, Mombasa, Kenya; 3 Department of Soil Science, Maseno University, Maseno, Kenya; 4 Department of Plant Sciences, Kenyatta University, Nairobi, Kenya; 5 Department of Microbiology, Kenyatta University, Nairobi, Kenya; 6 Department of Biological Sciences, Masinde Muliro University of Science and Technology, Kakamega, Kenya; Università Politecnica delle Marche, ITALY

## Abstract

Biological nitrogen fixation (BNF) in legumes plays a critical role in improving soil fertility. Despite this vital role, there is limited information on the genetic diversity and BNF of bacteria nodulating common bean (*Phaseolus vulgaris* L.). This study evaluated the genetic diversity and symbiotic nitrogen fixation of bacteria nodulating common bean in soils of Western Kenya. The genetic diversity was determined using 16S rRNA gene partial sequences while BNF was estimated in a greenhouse experiment. The sequences of the native isolates were closely affiliated with members from the genera *Pantoea*, *Klebsiella*, *Rhizobium*, *Enterobacter* and *Bacillus*. These results show that apart from rhizobia, there are non-rhizobial strains in the nodules of common bean. The symbiotic efficiency (SE) of native isolates varied and exhibited comparable or superior BNF compared to the local commercial inoculants (CIAT 899 and Strain 446). Isolates (MMUST 003 [KP027691], MMUST 004 [KP027687], MMUST 005 [KP027688], KSM 001 [KP027682], KSM 002 [KP027680], KSM 003 [KP027683] and KSM 005 [KP027685]) recorded equal or significantly higher SE (*p* < 0.05) compared to N supplemented treatments. The results demonstrate the presence of genetic diversity of native bacteria nodulating bean that are effective in N fixation. These elite bacterial strains should be exploited as candidates for the development of *Phaseolus vulgaris* inoculants.

## Introduction

Common bean (*Phaseolus vulgaris*) is one of the most important legumes in the human diet and serves as a significant source of proteins [1]. However, the yield of this crop among small holder farmers has been on the decline. The low productivity of Common been has been attributed to various factors including insect pests, diseases, drought, poor agronomic practices and plant nutritional deficiencies particularly nitrogen (N) [1]. The N fertilizers offer immediate solution to soil N deficiency and improve bean yields however these fertilizers are one of the most expensive agricultural inputs for smallholder farmers [2].

Therefore, such cheaper and environmentally friendly source of N should be sought to mitigate soil fertility problems [2, 3]. An important aspect of Common bean is its capacity to establish symbiotic associations with nodulating bacterial species that fix atmospheric nitrogen [4]. Nevertheless, symbiotic interactions between the crop and bacteria are not always effective in N fixation [4]. The ability to reduce atmospheric nitrogen to ammonia (N_2_ fixation) is only known among a limited number of bacterial species [5]. In the recent past N fixation has been shown to occur in many diverse bacterial representatives [6–8]. These representative bacterial strains include *Azotobacter*, *Bacillus*, *Enterobacter*, *Pseudomonas*, *Serratia*, *and Azospirillum* that are already being used as biofertilizers for enhancing the growth and yield of crops, as well as for maintaining soil fertility [9, 10]. Tropical soils of sub-Saharan Africa are known to harbor a great diversity of symbiotic bacteria despite pressure on the agricultural resources and harsh climatic conditions that adversely affect the soil ecosystem and biodiversity [11]. Studies in the last decade have indicated that the selection of effective strains adapted to local environmental conditions may represent a successful approach in boosting N fixation [12–14]. It is also important to assess the genetic diversity and estimate the symbiotic N fixation of indigenous bacterial community nodulating legumes in the main bean producing regions. The composition of the local strains has been reported to affect the response to inoculation with superior strains [4]. Unfortunately, there is limited knowledge on the genetic diversity and symbiotic N fixation of indigenous bacteria nodulating Common bean in most Kenyan ecosystems [11]. Increased interest in the utilization of symbiotic N fixing bacteria as biofertilizers in agriculture has prompted studies of their diversity. Improving our knowledge on the genetic diversity of symbiotic bacteria is important in understanding the role of biological N fixation in improving agricultural productivity. The aim of this study was therefore to determine the genetic diversity and estimate the symbiotic N fixation of indigenous bacteria nodulating Common bean in soils of Western Kenya.

## Materials and methods

Smallholder farmers in Western Kenya provided free access into their farms for this study.

### 2.1 Nodule sampling and isolation of root nodule bacteria

Seeds of common seeds were planted in farms at Kisumu (0° 05´35″ S, 0° 34° 41.32´´E) and Kakamega (0° 17′ 25.57″ N, 34° 45′ 50.02″) regions of Western Kenya. The planting was done in a randomized complete block design (RCBD). The plants were uprooted and fresh and red nodules were carefully removed from the roots of 100 representative flowering bean plants after 7 weeks of emergence across regions. The nodules were surface-sterilized in 1% NaOCl and rinsed in several changes of sterile water, and then crushed with a flame-sterilized blunt-tipped pair of forceps. A loopful of the crushed nodule suspension was streaked across the surface of Petri dish containing yeast extract mannitol agar (YEMA) media containing Congo red and incubated in the dark at 28°C. Single colonies were marked after 3 days and checked for purity by repeated streaking on YEMA medium and verifying a single type of colony morphology, absorption of Congo red (0.00125 mg kg^−1^), and a uniform Gram-stain reaction. Color, mucosity, margin, transparency, elevation and acid/alkaline reaction of the colony were evaluated on YEMA containing bromothymol blue (BTB) (0.00125 mg kg^−1^) as indicator. All the isolates were incubated at 28°C and stored at −20°C in 25% glycerol-YEM broth.

### 2.2 Authentication and symbiotic nitrogen fixation

The isolates were authenticated as root nodulating bacteria by reinoculating 1 mL of three-day-old pure YEM broth culture of the isolate on the host plant grown in a controlled environment in sterilized vermiculite in Leonard jar. The jars were arranged in randomized complete block design (RCBD) with four replications. The plants were watered with nitrogen-free nutrient solution. Treatments without inoculation and inorganic N fertilizer served as negative control while treatments without inoculation plus nitrogen fertilizer at a rate of 70 μg N mL−1 applied as KNO_3_ solution were used as positive control. The isolates were also compared with commercial rhizobia strain 446 and CIAT 899 as reference strains. After 45 days, shoot dry weight (SDW) was measured. Tissue N concentration per plant was analyzed using the Kjeldahl method and the N content per plant calculated by multiplying the SDW with the tissue N concentration [15]. Symbiotic efficiency (SE) was determined by comparing each isolate with N applied control (plant N content in inoculated pots/plant N content in N application) × 100 as previously described [16].

### 2.3 Total genomic DNA extraction

Single colonies of the authenticated isolates were picked and washed in 100 ul TE at pH 7.5 to obtain pelleted cells [17]. A volume of 250 ul of CTAB buffer was added to the washed pelleted cells; vortexed for 30 seconds, and incubated at 65°C for 15 minutes and then cooled to room temperature [17]. Then 250 ul of 24:1 chloroform: isoamyl alcohol was added to the samples and vortexed until the solution was homogenous with the suspension appearing white in colour. The suspension was then centrifuged for 10 minutes at 12000 rpm using a fixed angle rotor. The aqueous phase was transferred to a new sterile 1.5 ml microcentrifuge tube and equal amounts of cold isopropanol added and mixed gently [17]. The DNA was precipitated at -20°C for 30 minutes and centrifuged for 10 minutes at 12,000 rpm. The DNA was re-suspended in 30 ul TE buffer at pH 7.4 and the concentration and purity of the extracted DNA determined at 260 and 280nm using the Nanodrop Spectrophotometer [17].

### 2.4 PCR amplification and sequence analysis of the 16S rRNA gene

PCR amplification was done using the primer pair fD1 (5-AGAGTTTGATCCTGGCTCAG-3) and rD1 (5-AAGGAGGTGATCCAGCC-3), an approximately 1340 bp product specific to nearly full length of 16S rRNA gene [18, 19]. The PCR reaction was performed in a 30 μl volume containing Taq polymerase (pre-mix), 14.4 μl PCR water, 0.3 μl each of the forward and reverse primers and 0.5 μg template of DNA. Amplifications were carried out as follows: an initial denaturation at 95°C for 3 min followed by 35 cycles of denaturation at 94°C for 1min, annealing at 55°C for 1 min, extension at 72°C for 2 min and a final extension at 72°C for 3 min [19, 20]. Amplicons were resolved on a 1.5% agarose (1 X TBE, 90mM Tris pH 8.0, 90mM boric acid, 2mM EDTA) gel, stained with SYBR green, and visualized with UV light. The amplicons were purified using a QIAquick PCR Purification kit (QIAGEN Inc, CA) following manufacturer’s instructions, and the purified DNA samples were sequenced in both orientations using the ABI PRISM 377 DNA Sequencer (Applied Biosystems Inc, CA). Sequencing of the 16S PCR products was performed at Inqaba Biotech. (Pretoria, South Africa). The raw sequences were manually edited on Bioedit and Chomas Lite programs [21]. The obtained sets of sequences were deposited in GenBank under the accession numbers (KP02769-91 and KP137102-12).

### 2.5 Phylogenetic analysis

All sequences were checked for chimeric structures using the Mallard program [22]. A search for similar sequences using BLASTN against the National Center for Biotechnology Information (NCBI) database was performed, and sequence alignment between the query sequences and the identified nearest neighbours was performed using the CLUSTAL Omega program (http://www.clustal.org). A neighbour-joining tree of the aligned sequences was constructed (Saitou and Nei, 1987) using MEGA V6 [23, 24]. Evolutionary distances were computed using the Maximum Composite Likelihood method [25]. To obtain support values for the branches, bootstrapping was conducted with 1,000 replicates [26]. All sites, including gaps in the sequence alignment, were excluded pairwise in the phylogenetic analysis. Using the resultant neighbour-joining tree, each isolate’s sequence was assigned to a taxonomic group. The ribotypes were defined as those sequences sharing at least 98% sequence identity with each other [27].

## Results

### 3.1 Morphological characterization

The bacterial isolates obtained from the roots of bean plants grown across the two study sites were grouped according to their morphological and cultural characteristics. The isolates had entire colony margin and convex elevation. On YEMA media containing Congo red dye, the isolates either did not absorb the red dye or absorbed it lightly under incubation in the dark. Further, the isolates turned YEMA media substituted with bromothymol blue (BTB) into moderately yellow to deep yellow color and were thus considered to be acid producers and fast growers. The isolate colonies were creamy yellow, creamy white and milky white that were either opaque or translucent with smooth viscous or firm and dry texture. The isolates further produced a slimy material/extracellular polysaccharide (EPS) except the milky white colonies. The colony shapes were either circular or oval with diameters lying between 1 mm to 5.7 mm.

### 3.2 Affiliation of 16S rRNA gene sequences of the isolates

**Fig 1:**
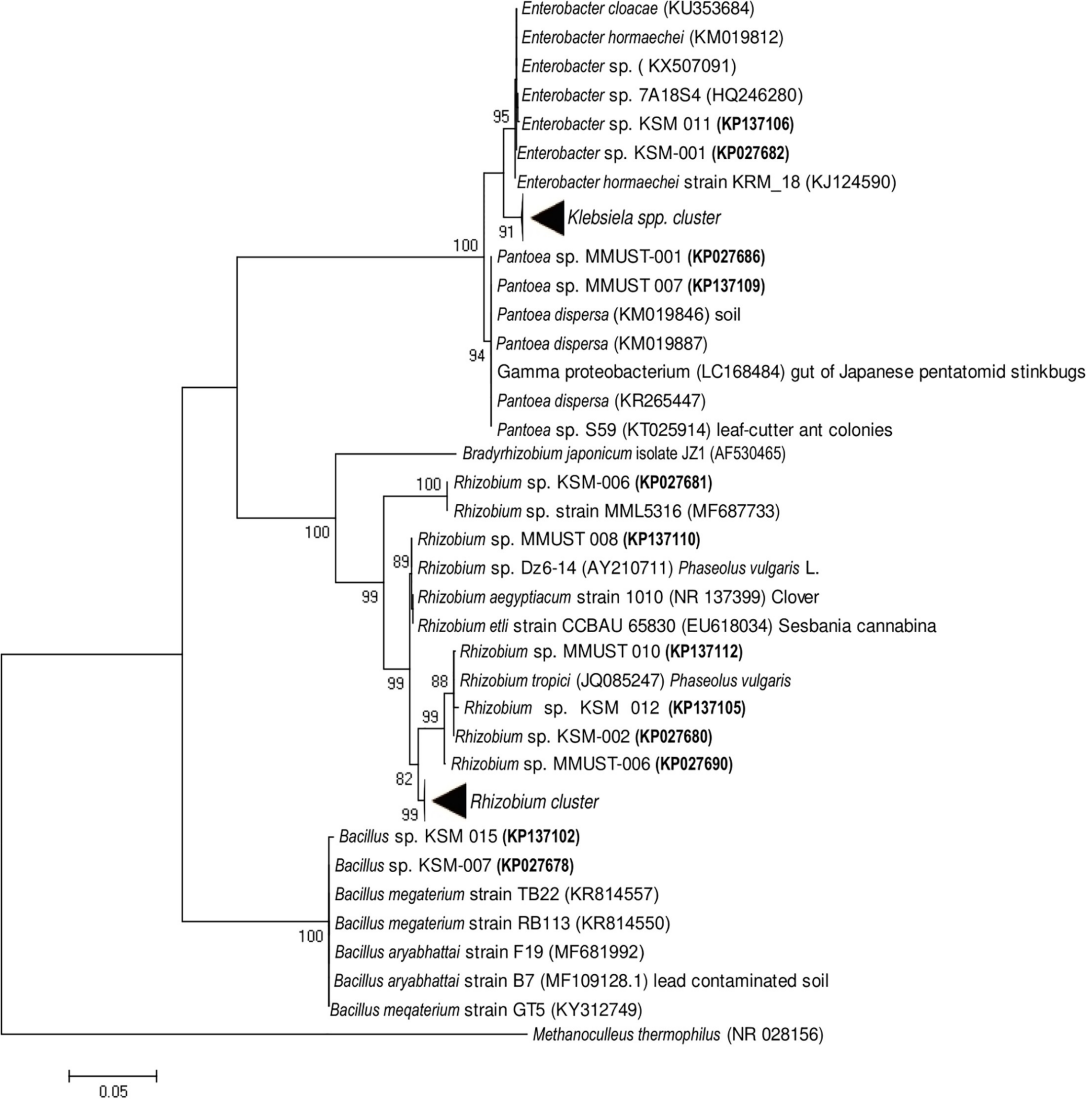
Phylogenetic tree of the 16S rRNA gene from 24 isolates (in bold) and closely related species. The sequence of *Methanoculleus thermophilus* (NR 028156) is included as an out group.

The PCR amplification of 16S rRNA genes of 25 selected isolates produced a single band of approximately 1500 bp. The sequences were deposited in the Genebank and assigned accessions numbers (Table 1). Comparison of our newly obtained 16S rRNA partial gene sequences with known bacterial sequences in the Genbank database using BLASTn analysis indicated sequence similarity of ≥ 99% (Table 1). Out of the total number of isolates, 32% were closely affiliated with members of the genus *Klebsiela* with >99% sequence identity. Two isolates (KSM-001 [KP027682] and KSM-011 [KP137106]) had >99% sequence identity with members of the genus *Enterobacter* while the other remaining two (MMUST-001 [KP027686] and MMUST-007 [KP137109]) had >99% sequence similarity with members of the genus *Pantoea*. The isolates that belonged to the three genera (*Klebsiela*, *Enterobacter* and *Pantoea*) formed one major cluster supported with a bootstrap value of 100% (Fig 1). Isolates (MMUST-002 [KP027689], MMUST-004 [KP027687], MMUST-005 [KP027688], KSM-003 [KP027683], KSM-005 [KP027685], KSM-008 [KP027684], KSM-009 [KP137108], and KSM-010 [KP137107]) were phylogenetically identical and had ≥99% sequence affiliation with *Klebsiella variicola* strain ALK036 (KC456523) and *Klebsiella* sp. N28 (KP410798). Isolates (KSM-001 and KSM-011) had a 100% sequence similarity with *Enterobacter hormaechei* strain D40 [KM019812]. Two isolates (MMUST-001 and MMUST-007) formed a sub-cluster with *Pantoea dispersa* [KM019887] supported with a bootstrap value of 96% (Fig 1). A total of 11 isolates also formed a major cluster belonging to the genus *Rhizobium* supported with a bootstrap value of 99%. Isolate KSM-006 [KP027681] and MMUST-008 [KP137110] were 100% affiliated with *Rhizobium* sp. strain MML5316 [MF687733]. Four isolates (MMUST-010 [KP137112], MMUST-006 [KP027690], KSM-002 [KP027680] and KSM-012 [KP137105]) formed a single sub-cluster supported with a bootstrap value of 99% and this sub-cluster was affiliated with strains of *Rhizobium tropici*. Five isolates (KSM-004 [KP027679], KSM-013 [KP137104], KSM-014 [KP137103], MMUST-003 [KP027691] and MMUST-008 [KP137110]) formed a sub-cluster (*Rhizobium* cluster) supported with a bootstrap value of 100% and was affiliated to strains belonging to *Rhizobium leguminosarum* (*R*. *leguminosarum* strain Vaf-23 [KF662887], *R*. *leguminosarum* strain INTA D156 [KX0660640]. Two isolates (KSM-007 [KP027678] and KSM-015 [KP137102]) formed a major cluster with several species belonging to the genus *Bacillus*. This was supported by a bootstrap value of 100%. These two isolates were affiliated with *Bacillus aryabhattai* [MF109128] and *Bacillus megaterium* [KY31279] with ≥99% sequence identity. The evolutionary relationship estimated using matrix pairwise genetic distances for the 16S rRNA partial gene sequences indicated that the isolates were closely related (Table 2). The longer genetic distance of 0.26 was observed between the genus *Bacillus* (KSM 007[KP027678]) and *Pantoea* (MMUST 001[KP027686]). Similar evolutionary distances were observed between isolates (KSM 007[KP027678]) and (KSM 001[KP027682]).

**Table 1:** Taxonomic affiliation and percentage sequence similarities of native isolates with closest relatives from the Genbank database.

Isolate ID	Accession No.	Closest taxonomic sp. affiliation	Source	Sequence Similarity (%)
KSM 001	KP027682	*Enterobacter hormaechei* KRM_18 (KJ124590)	Rhizospheric soil	100
KSM 002	KP027680	*Rhizobium tropici* RP261 (DQ406713)	*Phaseolus vulgaris*	99
KSM 003	KP027683	*Klebsiella variicola* NGB-FR96(LC049192)	Faba bean	100
KSM 004	KP027679	*Rhizobium leguminosarum* RMCC TP06122 (KY587870)	Clover	100
KSM 005	KP027685	*Klebsiella variicola* NGB-FR116 (LC049205)	Faba bean	99
KSM 006	KP027681	*Rhizobium sp*. GGC2 (KF008226)	*Vigna mungo*	100
KSM 007	KP027678	*Bacillus aryabhattai* FJAT-40026 (MG905898)	Soil	100
KSM 008	KP027684	*Klebsiella sp*. NB-90 (KC455417)	Sugarcane	100
KSM 009	*KP137108*	*Klebsiella sp*. D3S(GU259534)	*Drosera burmannii*	99
KSM 010	*KP137107*	*Klebsiella variicola R5-431* (JQ659780)	*Jatropha curcas*	99
KSM 011	*KP137106*	*Enterobacter hormaechei* IARI-NIAW2-34(KF054945)	Wheat rhizospere	100
KSM 012	*KP137105*	*Rhizobium tropici* NS-10(KU305702)	Peanut	100
KSM 013	*KP137104*	*Rhizobium sp*. *vd6* (KX898585)	Legumes	100
KSM 014	*KP137103*	*Rhizobium leguminosarum* RMCC TP4321 (KY587906)	Clover plants	100
KSM 015	*KP137102*	*Bacillus aryabhattai* LS9 (GU563346)	Rhizosphere soil	99
MMUST 001	KP027686	*Pantoea dispersa* g58 (KM019887)	Soil	100
MMUST 002	KP027689	*Klebsiella sp*. Gad1 (KJ940119)	*Arachis hypogaea*	99
MMUST 003	KP027691	*Rhizobium leguminosarum* SWD14-4 (KJ634554)	*Pisum sativum*	100
MMUST 004	KP027687	*Klebsiella variicola* NGB-FR116(LC049205)	Faba bean	100
MMUST 005	KP027688	*Klebsiella variicola* DPMH (JX968498)	Barley	99
MMUST 006	KP027690	*Rhizobium tropici* CPAO 29.8 (EU488739)	*Phaseolus vulgaris*	99
MMUST 007	*KP137109*	*Pantoea dispersa* M1R4 (GQ246183)	cultivated crops	100
MMUST 008	*KP137110*	*Rhizobium sp*. ESC1110 (KF638350)	*Phaseolus vulgaris*	100
MMUST 009	*KP137111*	*Rhizobium leguminosarum* Vaf-23 (KF662887)	*Vavilovia formosa*	99
MMUST 010	*KP137112*	*Rhizobium tropici strain* CIAT 899 (NR_102511)	*Phaseolus vulgaris*	99

**Table 2:** Genetic distance among representative native bacterial isolates nodulating common bean.

Bacterial Strains	1	2	3	4	5	6	7	8	9	10	11	12	13	14
*P*. *dispersa* MMUST 001 (KP027686)														
*E*. *hormaechei* KSM 001 (KP027682)	0.02													
*K*. *variicola* MMUST 004 (KP027687)	0.03	0.002												
*K*. *variicola*(MMUST 005 (KP027688)	0.03	0.02	0.00											
*K*. *variicola* KSM 003 (KP027683)	0.03	0.02	0.00	0.00										
*K*. *variicola* KSM 005 (KP027685)	0.03	0.02	0.00	0.00	0.00									
*Rhizobium* sp KSM 008 (KP027684)	0.03	0.02	0.00	0.00	0.00	0.00								
*Klebsiella* sp MMUST 002 (KP027689)	0.03	0.02	0.00	0.00	0.00	0.00	0.00							
*R*. *tropici* MMUST 006 (KP027690)	0.22	0.22	0.23	0.23	0.23	0.23	0.23	0.23						
R. leguminosarum MMUST 003 (KP027691)	0.22	0.23	0.23	0.23	0.23	0.23	0.23	0.23	0.02					
R. leguminosarum KSM 004 (KP027679)	0.22	0.23	0.23	0.23	0.23	0.23	0.23	0.23	0.02	0.00				
*R*. *tropici* KSM 002 (KP027680)	0.22	0.22	0.23	0.23	0.23	0.23	0.23	0.23	0.00	0.02	0.02			
*Rhizobium* sp KSM 006 (KP027681)	0.23	0.24	0.24	0.24	0.24	0.24	0.24	0.24	0.06	0.06	0.06	0.06		
*B*. *aryabhattai* (KSM 007 (KP027678)	0.26	0.26	0.25	0.25	0.25	0.25	0.25	0.25	0.12	0.12	0.12	0.12	0.22	

### 3.3 Authentication and assessment of symbiotic nitrogen fixation

All the isolates except two initiated nodulation on the host crop and were thus considered as legume nodulating bacteria. The nodules formed were pink and the leaves of the nodulated plants were dark-green, while uninoculated and unfertilized control plants turned yellow after 21 days. In contrast, control plants grown with and without nitrogen supplementation did not form nodules. Analysis of the SDW and N content of the inoculated bean plants revealed a wide range of variation in symbiotic N fixation of the isolates (Tables 3 and 4). Representative isolates (MMUST 003[KP027691], MMUST 004[KP027687] and MMUST 005[KP027688]) were significantly (*p* < 0.05) effective in N fixation compared to the reference strains (*CIAT 899* and *Strain 446*). Similarly, CIAT 899 recorded signifcantly lower SE (p < 0.05) compared to all the isolates from Kisumu county (Table 2).

**Table 3:** SDW, N concentration and SE of representative isolates in Kakamega, Kenya.

Isolate ID	Strain affiliation	Sampling Location	SDW	N Content	SE (%)
*MMUST 003*	*R*.*leguminosarum (KJ634554)*	Kakamega County	1.76a	2.33b	100.0b
*MMUST 004*	*Klebsiella variicola (LC049205)*	Kakamega County	1.78a	2.48b	107.0b
*MMUST 005*	*Klebsiella variicola (JX968498)*	Kakamega County	1.85a	3.80a	164.0a
*MMUST 006*	*Rhizobium tropici (EU488739)*	Kakamega County	1.73a	1.80bc	78.0bc
Reference	Strain 446	Kenya	1.69a	2.56b	110.0b
Reference	CIAT 899	Kenya	1.50a	1.55bc	67.0bc
+VE Control	N supplemented	Commecial	1.68a	2.32b	100.0b
-VE Control	Non N supplemented	Commercial	1.63a	0.75c	-
LSD (5%)			0.39	1.13	49

LSD: Least Significant Difference of means; SE: Symbiotic Efficiency; Means within a column followed by the same letter (s) are not significantly different at p<0.05

**Table 4:** SDW, N concentration and SE of representative isolates in Kisumu, Kenya.

Isolate ID	Taxonomic affilitaion	Sampling Location	SDW	N Content	SE (%)
*KSM 001*	*E*. *hormaechei (KJ124590)*	Kisumu County	1.59bc	2.89abc	125.0abc
*KSM 002*	*Rhizobium tropici (DQ406713)*	Kisumu County	1.52c	2.31bc	100.0bc
*KSM 003*	*K*. *variicola (LC049192)*	Kisumu County	1.84bc	3.94a	170.0a
*KSM 004*	*R*. *leguminosarum (KY587870)*	Kisumu County	1.64bc	2.07bcd	89.0bcd
*KSM 005*	*K*. *variicola (LC049205)*	Kisumu County	2.01a	3.01ab	130.0ab
*KSM 006*	*Rhizobium sp*. *(KF008226)*	Kisumu County	1.65ab	1.72bcd	74.0bcd
*KSM 007*	*B*. *aryabhattai (MG905898)*	Kisumu County	1.65bc	2.17bcd	94.0bcd
*KSM 008*	*Klebsiella sp*. *(KC455417)*	Kisumu County	1.88ab	1.89bcd	81.0bcd
Reference	Strain 446	Commercial	1.69abc	2.56abc	110.0abc
Refrence	CIAT 899	Commercial	1.50c	1.55cd	67.0cd
+VE Control	N supplemented		1.67abc	2.32bc	100.0bc
-VE Control	Non N supplemented		1.63bc	0.75d	-
LSD (5%)			0.36	1.43	62.0

LSD: Least Significant Difference of means; SE: Symbiotic Efficiency; Means within a column followed by the same letter (s) are not significantly different at p<0.05

Across the sites, the isolates(MMUST 005[KP027688], KSM 001[KP027682], KSM 003[KP027683] and KSM 005[KP027685] recorded higher SE compared to *Strain 446*. Of the reference strains, CIAT 899 recorded the least symbiotic N fixation across the two regions.

## Discussion

### 4.1 Morphological characterization

Morphological and cultural growth characteristics of bacteria nodulating different legume crops has widely been confirmed using YEMA media [28]. Our isolates absorbed Congo red dye and showed a Gram negative reaction during incubation in the dark. These characteristics are typical traits of root nodule bacteria from legumes [29]. Further, the isolates turned YEMA media substituted with bromothymol blue (BTB) into moderately yellow to deep yellow color indicating that they are fast growers and acid producers [28]. Most of the isolates produced copious amount of extracellular polysaccharide (EPS) that is considered an adaptive feature for bacteria against temperature, salinity, and pH fluctuations in the soil. Nodulating bacteria with the ability to withstand such environmental stresses could be suitable candidates for the development of commercial legume inoculants. The range in the diameters of the colonies with regular and circular margins observed in this study are similar to those previously reported [30]. The variation in the morphological characteristics of the isolates could be an indication of diverse indigenous bacteria nodulating Common bean.

### 4.2 Affiliation of 16S rRNA partial gene sequences

The amplification of the 16S rRNA partial gene sequences the isolates using specific primers generated a single band of approximately 1500 base pairs. This is usually the approximate fragment size of this target gene that is commonly used for bacterial identification [31]. The single fragment shows that the 16S rRNA gene is a conserved region within the bacterial genome. The degree of conservation observed in the 16S rRNA gene is due to its importance as a critical component of cell function [32]. As a result of high level of conservation, the 16S rRNA gene is often serves as a marker for taxonomic and phylogenetic analysis [33]. Although the absolute rate of change in the 16S rRNA gene sequence still remains unknown, it marks evolutionary distance and relatedness of organisms [34, 35].

The 16S rRNA partial gene sequences analysis placed the 25 isolates into 5 genera consisting of *Pantoea*, *Klebsiella*, *Rhizobium*, *Enterobacter* and *Bacillus*. These clusters suggest the high level of diversity among the isolates and further confirms the reliability of 16S rRNA gene sequences in establishing genus affiliation [36]. Based on partial 16S rRNA gene sequence analysis, the five genera identified demonstrate that the root nodules of Common bean are occupied by a diverse group of bacteria. In addition, the results demonstrate that apart from rhizobia, there are phylogenetically diverse bacterial species nodulating beans. In terms of relative abundance and diversity, the non-rhizobial bacteria were higher in the bean nodules. Similarly, different authors have reported the presence of non rhizobia bacteria in the nodules of legumes [37, 38]. Lu et al. (2017) and De Meyer et al. (2015) argued that legume nodules represent a unique ecological niche that can accommodate any compatible soil microbes. However, these results are contrary to those previously described [11, 39]. These authors considered nodule isolates lacking typical Rhizobia growth characteristics as contaminants.

The five groups belonging to genera *Pantoea*, *Bacillus*, and *Enterobacter* have previously been isolated from the nodules of different legume crops [37, 38, 40]. Reports have attributed the occurrence of different strains in the nodules to multiple symbiotic relationships in the particular region of isolation [40, 41]. The genetic distance of the isolates ranged from 0.00 to 0.26, revealing least and highest level of differentiation. Lack of genetic distance between isolates has been attributed to a common ancestry with minimum recombination rates [42, 43]. Overall, our results indicate high nodulation promiscuity of *P*. *vulgaris* with diverse N fixing bacteria in soils of Western Kenya. Reports have shown that the use of fertilizers, pesticides and herbicides can alter bacterial diversity in different agro ecological zones with different cropping history [44, 45]. Phylogenetic analysis of symbiotic genes is essential in providing information on the symbiotic properties of N fixing bacteria and not merely for the description of new strains [46].

### 4.3 Authentication and symbiotic nitrogen fixation

Most of the isolates in the five genera initiated nodulation on the host crop except (MMUST-001 [KP027686] and MMUST-002 [KP027689]) and were considered bean nodulating bacteria. The two isolates that failed to initiate nodulation were closely affiliated with *Pantoea dispersa* g58 (KM019887) and *Klebsiella* sp. Gad1 (KJ940119) and have previously been isolated from soil and nodules, respectively. Nodulation is considered as a confirmatory test for bacteria nodulating legumes (BNL). Several authors have demonstrated that no bacterial isolate can be regarded as BNL until its identity has been confirmed through plant infection test on an appropriate host crop [47, 48]. Notably, the Genus *Klebsiella* that failed to nodulate in Kakamega initiated nodulation in Kisumu. Similarly, other studies have reported that bacterial isolates from soils in different geographical locations failed nodulation test but were later confirmed to be BNL [49]. Failure to initiate nodulation in the host crop has been attributed to loss of plasmids or genes responsible for nodule formation and N fixation [50]. Our results demonstrate that nodulation test alone should not be used as a confirmatory test for BNL. Nodules were not formed in the plants grown with or without nitrogen supplementation indicating lack of external contamination during the experiments. Lack of contamination is considered as a requirement in BNL authentication experiment [19, 51].

All the five genera of the native bacteria were able to nodulate and fix N with *P*. *vulgaris* in soils of Western Kenyan soils. The differences in the SDW and N content of the plants inoculated with different strains of native bacteria indicate their variation in symbiotic N fixation. The varaition in N fixation among genera has been attributed to the differences in the chromosal or plasmid borne symbiotic genes [36]. For example, in Kakamega, isolates (MMUST 003 [KP027691], MMUST 004 [KP027687] and MMUST 005 [KP027688]) recorded a comparable or superior symbiotic N fixation compared to the commcerial inoculants (CIAT 899 and Strain 446). Similarly, isolates (KSM 001 [KP027682], KSM 002 [KP027680], KSM 003 [KP027683] and KSM 005 [KP027685]) in Kisumu exhibited better N fixation characteritics compared to the commercial inoculants. These results support those of Kawaka et al. (2014) and Mwenda et al. (2018), who isolated native bacteria from bean nodules with higher symbiotic N fixation in Kenya. The presence of superior native N fixing bacteria in soils Western Kenya could be exploited as candidate strains to enhance bean inoculation programmes. Since the isolates are from two different locations across Western Kenya, they could be well adapted to diverse local soils and climatic conditions.

## Conclusions

The results of this study have demonstrated that soils across different regions in Western Kenya harbour diverse indegenous symbiotic bacteria that initiate nodulation in common bean apart from the rhizobia. These native bacterial isolates exhibited comparable or superior symbiotc N fixation characteristics compared to the locally available commercial inoculants for *Phaseolus vulgaris*.

Notably, it was established that most of the symbiotic strains recovered are not known to commonly nodulate *P*. *vulgaris*. These elite isolates should be subjected to further investigations under different environmental conditions to optimize their N fixing potentials.
